# Object numerosity influence sensorimotor programs evoked by graspable object nouns

**DOI:** 10.3389/fpsyg.2026.1770533

**Published:** 2026-02-12

**Authors:** Gioacchino Garofalo, Elena Gherri, Luisa Lugli, Valter Prpic

**Affiliations:** 1 Department of Philosophy, University of Bologna, Bologna, Italy; 2 Department of Theoretical and Applied Sciences, eCampus University, Novedrate, Italy

**Keywords:** affordances, embodied cognition, grasp compatibility, nouns, numerosity

## Abstract

Grasping actions can be evoked and modulated by a range of linguistic and abstract concepts, including object nouns, adjectives and numbers. The present study investigated whether the grasp-compatibility effect—typically elicited by graspable object nouns - is sensible to variations in numerosity. In Experiment 1, we tested graspable object nouns presented in the singular vs. plural form. In Experiment 2, we enhanced the salience of numerosity by adding quantifiers (one vs. many) to the same nouns. We hypothesized that object numerosity would be encoded alongside object size, thereby modulating the grasp-compatibility effect. Specifically, we expected that single object nouns, which are compatible with specific grasping actions, elicited a grasp-compatibility effect. Such effect should no longer be elicited when multiple objects are evoked (e.g., *one apple* is compatible with a power grasp., whereas *many apples* is no longer compatible with the same grasping action). The results indicate that the grasp-compatibility effect is not modulated by grammatical numbers alone (Experiment 1), but it disappears when numerosity is made explicit through the quantifier “many” (Experiment 2). These findings suggest that object numerosity can influence the sensorimotor simulation elicited by graspable object nouns, but only when numerosity is explicitly conveyed through semantic quantification rather than grammatical morphology.

## Introduction

According to the embodied cognition framework (Barsalou, 2003, 2008; Borghi and Binkofski, 2014; Gallese, 2003; Gallese and Cuccio, 2018; Gallese and Lakoff, 2005; Pulvermüller, 2018; Taylor and Zwaan, 2009), cognitive processes are deeply rooted in the body’s interactions with the environment. This framework challenges the classical approach to cognition (Fodor, 1975; Pylyshyn, 1984, 2003) in which cognitive processes are disengaged from sensory and motor experiences. By contrast, embodied theories propose that cognitive processes are grounded in sensory, motor and even emotional systems. A general principle underlying this approach is that cognitive processes, such as numerical cognition or language, recruit neural systems that were originally engaged during direct experience of events in the world during direct experience with the event in the world (Anderson, 2010; Gallese, 2008).

Central to this perspective is the concept of affordances, originally introduced by Gibson (1979), which refers to the action possibilities that objects offer to an agent based on their physical properties and the agent’s motor capabilities. For example, the possibility to interact with handles by grasping them is directly perceived by an agent capable of such an action. Rather than inferred through representational processing (Gibson, 1979). More recently, Ellis and Tucker (2000) extended this notion by introducing the concept of micro-affordances, referring to the specific motor actions component (such as the wrist orientation, or a specific grip) evoked by an object’s intrinsic properties such as size, shape, and orientation. For example, viewing a small object like a strawberry potentiates a precision grip, whereas a larger object like an apple evokes a power grasp. These Perception-action links have been widely demonstrated in studies using the grasp-compatibility task (e.g., Ellis and Tucker, 2000; Bub and Masson, 2010). This task requires participants to categorize an attribute of a single, visually presented object by performing a power or precision grip. Typically, participants’ performance improves when the response grip is compatible with the movement usually performed to interact with the object’s size/shape (compatible trials, i.e., apple and power grip), and it worsens when the response grip is incompatible (incompatible trials, i.e., apple precision grip). Studies in which multiple objects were presented in the visual scene, investigating the competition between potential interactions evoked by those objects, showed improved target identification performance when target and distractor evoked similar affordances (grasp size or handle orientation) compared to when their affordances were dissimilar (e.g., Ellis et al., 2007; Pavese and Buxbaum, 2002).

Notably, the grasp-compatibility effect (GCE) can also be evoked by the noun of graspable objects (e.g., Tucker and Ellis, 2004; Marino et al., 2014), suggesting that the online availability of visual information is not a mandatory requirement for this effect. Furthermore, the GCE elicited by object nouns can be modulated by the presence of other words, such as adjectives (Garofalo et al., 2021, 2024b). For example, in a grasp-compatibility task in which an adjective was paired with the object noun, the GCE was eliminated or even reversed when the adjective denoted an object property hindering the possibility of physical interaction with the object (e.g., broken, hot; “disadvantageous adjectives”; Garofalo et al., 2024b; Garofalo et al., 2021). By contrast, when the noun was combined with an adjective specifying a manipulable feature of the object (e.g., shape), the GCE emerged for both natural and artifact object categories, whereas color adjectives resulted in GCEs selectively for natural objects but not for artifacts (Garofalo et al., 2021). Collectively, these findings reveal that sensorimotor simulation during language processing is compositional, extending beyond isolated words to semantic integration across multiple elements. This evidence supports a dynamic account of language comprehension wherein meaning emerges through the sensorimotor integration of multiple linguistic constituents, that can dynamically update the affordances evoked by object nouns (Garofalo et al., 2021, 2024b; Pulvermüller, 2002; Santana and De Vega, 2013; Visani et al., 2022).

According to the Grounded, Embodied, and Situated cognition (GES) framework (Fischer, 2012), words share a common representational platform with numbers, which are grounded in space. Over the past decades, evidence for this has come from a variety of different paradigms. For instance, grammatical numbers elicit compatibility effects that are also found with digits (Roettger and Domahs, 2015). Consistent with the direction of the mental-number-line (i.e., small numbers on the left, large numbers on the right; Restle, 1970), responses to singular words are faster when executed by the left compared to the right hand, while the opposite pattern is observed for plural words. In addition, when Lachmair et al. (2014) presented words referring to objects typically found in upper or lower vertical space (e.g., roof vs. root), preceded by a digit (1, 2, 8, or 9) in a lexical decision task, they demonstrated that high numbers, typically associated with the upper space, can facilitate lexical access to words compatible with the upper space, such as bird. A follow-up study by Lachmair et al. (2018) showed that the number-to-word priming effect between numbers and words related to physical space (e.g., roof vs. root) can be further modulated by grammatical numbers (singular vs. plural forms). Further support for the common representation of numbers and words came from a recent study by Varma et al. (2024). They demonstrated that graded words (words that vary by degree on a scale, e.g., “calm,” “annoyed,” “angry,” “furious”) elicit numerical processing effects similar to those observed in numerical tasks, suggesting that magnitude representations activated by numbers are also recruited during the processing of word gradations.

Numerical magnitude influences the planning and execution of hand actions. For example, Andres et al. (2004) reported electromyographic evidence showing that grip closure is initiated faster in response to small digits, whereas grip opening is initiated faster in response to large digits. This initial observation was corroborated by behavioural evidence showing the modulatory effect of numbers on grasping actions (Lindemann et al., 2007), with precision grip actions initiated faster in response to small numbers and power grips initiated faster in response to large numbers. Interestingly, responding to large numbers resulted in increased maximum grip aperture regardless of grip (power vs. precision). These findings were further replicated and extended by Moretto and di Pellegrino (2008), who observed an advantage for precision/power grip responses based on number magnitude both during semantic (parity; i.e., odd vs. even) and non-semantic (colour; i.e., red vs. blue) number processing. This latter finding suggests that processing surface characteristics of symbolic numbers can automatically prime grasping gestures (see also Namdar et al., 2014 for similar conclusions), analogously to what observed with object size. Crucially, the mere observation of hand actions can influence number processing, suggesting that the link between numbers and grasping is bidirectional (Badets and Pesenti, 2010, 2011; Ranzini et al., 2022). In addition, perceiving graspable objects with clear action affordances can interfere with numerical processing (Ranzini et al., 2011). When graspable objects preceded numerical stimuli, faster responses were observed for small rather than to large numbers. This is likely due to the fact that only small quantities are compatible with grasping actions, suggesting that number processing and object affordances involve overlapping mechanisms. From a theoretical point of view, most studies linking hand actions and number processing support the existence of a common cognitive code for representing numbers and physical quantities within a generalized magnitude system devoted to action planning (Bueti and Walsh, 2009; Walsh, 2003).

Although previous studies have explored how grasp-related affordances are modulated by linguistic context and how numerical magnitude interacts with action representations, little is known about how these two domains, language-related sensorimotor simulation and numerical magnitude, jointly shape sensorimotor simulation during word comprehension. In particular, while research GCE has mainly focused on the semantic features of single objects or adjective-noun combinations, the role of object numerosity as a linguistic cue to quantity has so far received little attention. Understanding whether and how numerosity modulates the GCE can provide crucial insights into the specificity and flexibility of embodied semantic representations (Borghi and Riggio, 2015; Mazzuca et al., 2021; Pulvermüller, 2018; Van Dam et al., 2012; Willems and Casasanto, 2011), revealing whether the motor system encodes not only what an object is, but also how many instances of it are implied by language.

The evidence discussed above suggests the existence of a complex interplay between language, numbers and action planning. In the present study we investigated the impact of object numerosity on the sensorimotor simulation elicited by language processing in a grasp-compatibility task. Participants were instructed to categorize objects’ nouns as natural or artifact by performing a reach to grasp action using a power or precision grip. In Experiment 1, and in line with previous studies (Lachmair et al., 2018; Roettger and Domahs, 2015), we manipulated numerosity using the singular or plural forms of graspable object nouns (e.g., cherry vs. cherries). In Experiment 2, we further enhanced the sense of numerosity by adding quantifiers to the same stimuli used in Experiment 1 (e.g., one cherry vs. many cherries). If object numerosity is encoded together with object size, we expect to observe GCE modulations by object numerosity. Specifically, the GCE should be reduced or even eliminated when the object noun denoted a multitude of items (e.g., “apples” in Experiment 1 or “many apples” in Experiment 2), because the motor program required by the task (grasping a single object with a power or a precision grip) is no longer compatible with the action of grasping multiple objects.

## Experiment 1

### Method

#### Participants

We conducted an a-priori power analysis based on the interaction effect between Grasp-compatibility and Adjective from Garofalo et al. (2021) (Experiment 2). We tested the effect size scenarios ranging from 100% to 40% on coefficients from a linear mixed model to account for potential attenuation due to additional experimental factors. While 10 participants would suffice at full effect size, we determined that 50 participants were necessary to achieve approximately 89% power under the most conservative scenario (i.e., 40%). Fifty-one individuals volunteered for the experiment at the University of Bologna. Three participants were subsequently excluded from the analyses since they were left-handed. All the remaining participants (40 defined themselves as female, 8 as male; mean age = 21.8 ± 3.7 years) were right-handed according to a standard handedness inventory (Oldfield, 1971), native Italian speakers, and had normal or corrected-to-normal vision. All were at least 18 years old at the time of the study and were naïve to its purpose. Informed consent was obtained from all participants prior to their participation, in accordance with the ethical standards of the Declaration of Helsinki. The study received approval from the local ethics committee (approval number: 0026467).

#### Apparatus and stimuli

**Figure 1:**
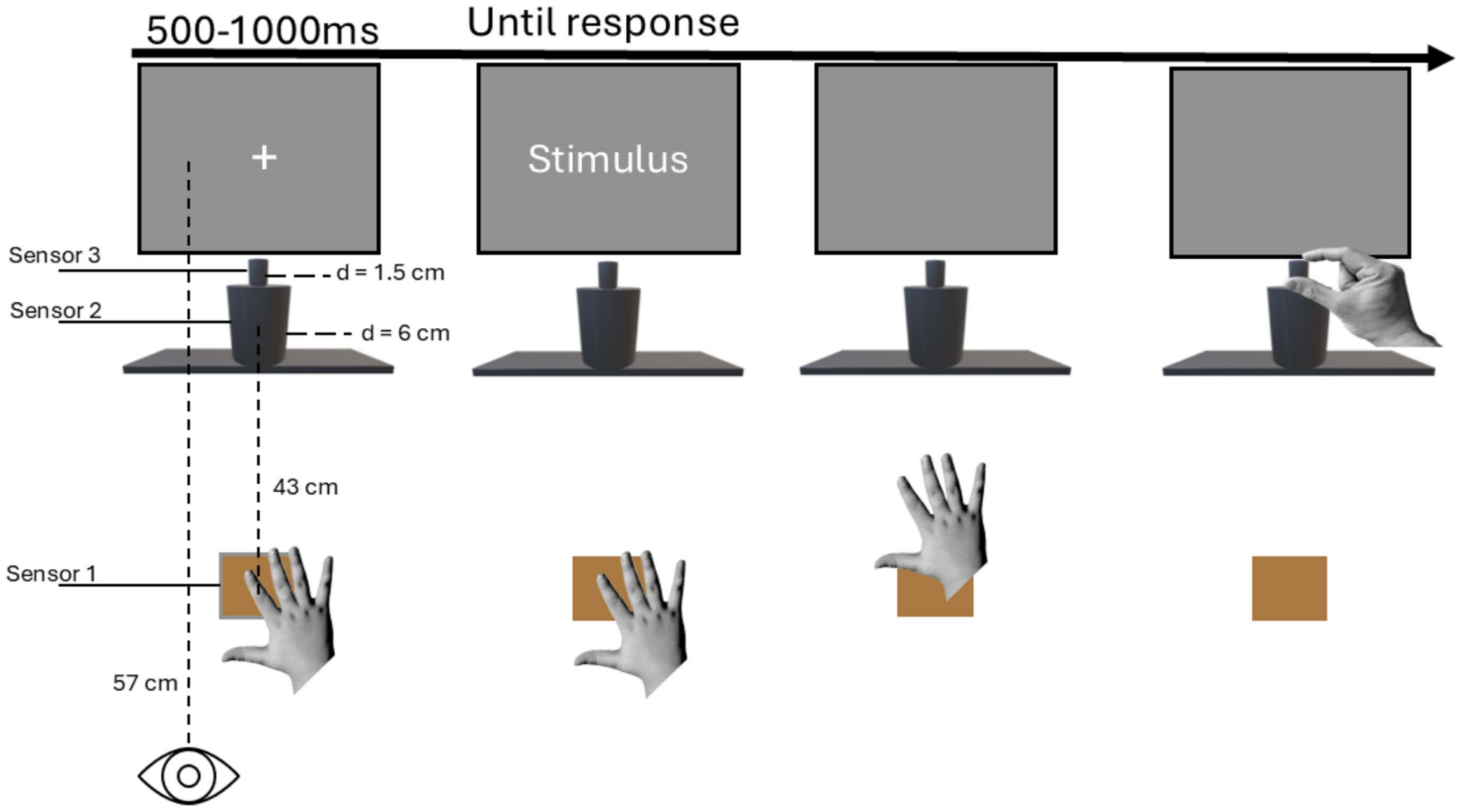
Representation of the experimental setup and the sequence of events.

The experiment was conducted in a sound-attenuated, dimly lit room. The experimental setup included a 24″ monitor connected to a computer running E-Prime 2.0 software. Viewing distance was maintained at 57 cm using an adjustable head-and-chin rest positioned in front of the screen. The response device was comprised of three components (see Figure 1): two wooden cylinders stacked vertically and a square starting base (10 cm × 10 cm). The lower cylinder’s dimensions were suited for a power grasp (height = 14 cm, diameter = 6 cm), while the upper cylinder’s dimensions were suited for a precision grasp (height = 4 cm, diameter = 1.5 cm). A capacitive sensor was attached to each cylinder as well as the starting base. The cylinders were positioned 43 cm from the chin rest, and the starting base was centered relative to the participants’ body midline (see Figure 1). All three components of the response device were connected to an external USB device trigger via separate capacitive sensors. This configuration enabled measurement of Reaction Times (RTs) and Movement Times (MTs) for both precision and power reach-to-grasp movements (Bub and Masson, 2010; Makris et al., 2011; Symes et al., 2008). RTs and MTs correspond to the planning and execution (or actualization) phases of movement, respectively (Castiello, 1999, 2005; Glover, 2004; Jeannerod, 1984). Each trial began when participants placed their right palm on the starting base. This design prevented activation of hand or arm muscles, thereby reducing potential motor interference between the key-press action and the response action. Upon hand placement on the starting base, a fixation cross (bold Courier New, 30-point) appeared at the screen’s center. To prevent habituation, the fixation cross remained visible for a randomly selected duration between 500 and 1,000 ms. Following these variable intervals the object noun (in its singular or plural form) replaced the fixation cross and remained displayed until the participant lifted their hand from the resting position. Stimuli appeared at screen’s center (1920 × 1280 resolution) in white text on a black background (bold Courier New, 24-point).

The stimuli consisted of 32 Italian object nouns. Sixteen nouns denoted natural objects: eight graspable with a precision grip (e.g., “oliva,” olive) and eight with a power grip (e.g., “patata,” potato). Sixteen nouns denoted artifacts: eight graspable with a precision grip (e.g., “chiodo,” nail) and eight with a power grip (e.g., “bottiglia,” bottle). Across 256 trials, each noun can be equally presented in its singular or plural form. In 32 catch-trials, nouns were substituted by pseudowords (e.g.,“tapata”). Each pseudoword was presented randomly with two repetitions each.

#### Procedure

Participants were instructed to categorize object nouns as either natural or artifact. They performed either a right-hand precision or power reach-to-grasp movement toward the custom-made response device (see Figure 1). Grip movements were either compatible or incompatible with those typically used to manipulate the objects denoted by the nouns (e.g., precision grasp compatible with olive but incompatible with bottle). Half the participants categorized natural nouns using whole-hand (power) reach-to-grasp movements and artifact nouns using index-thumb (precision) reach-to-grasp movements. The remaining participants followed the reverse category-response mapping. With object nouns participants performed the categorization task (reach trials). Participants were instructed to withhold reach-to-grasp movements and simply lift their right hand when pseudowords were presented (catch trials). Catch trials were randomly interspersed throughout the task to ensure participants semantically processed the noun. On all trials (both reach and catch trials), stimuli disappeared from the screen when participants lifted their hand.

The experimental task (288 total trials) started after a 40-trial practice session. Participants were tested individually and instructed to optimize the balance between speed and accuracy while performing the task.

#### Analyses

Participants’ error rates, RTs (ms), and MTs (ms) were recorded and analyzed. Practice and catch trials were excluded from analyses. RTs were measured from stimulus onset until the moment participants lifted their hand from the starting base. MTs were calculated as the time difference between completion of the reach-to-grasp movement (grasping one of the two cylinders) and hand lift from the starting position.

Correct RT and MT data were modeled after excluding anticipatory responses (RTs < 150 ms). The remaining RT distributions were examined for deviations from normality. Since these distributions typically exhibit positive skewness, we applied an iterative Box-Cox procedure to identify the optimal lambda transformation parameter (Box and Cox, 1964; Klein Entink et al., 2009) that best reduced skewness across participants. Subsequently, transformed RTs exceeding 3 SD were excluded from further analyses.

Given the experimental design with multiple observations per participant and stimulus, we employed a mixed-model approach for data modeling. All models in the study included fixed effects of Grasp-Compatibility (2 levels: compatible and incompatible), and Form (2 levels: Singular and Plural), along with their interactions. Participants and Stimuli were designated as random effects (Barr et al., 2013). Trial errors were analyzed using logistic regression, while RTs and MTs were analyzed using linear mixed models (LMM). All models were computed in R 4.2.0 (R Core Team, 2022) using the lmer() function from the lme4 package (Bates et al., 2015). We calculated 95% confidence intervals while accounting for participant-by-participant variation, interpreting non-overlapping intervals as evidence of differences between conditions.

### Results

Errors: The error rate data showed that participants made categorization errors on 2.3% (567 datapoints) of the total number of trials. Individual participants showed error rates ranging between 0 and 3.1% of all trials. The model did not show any reliable main effect or interaction.

**Figure 2:**
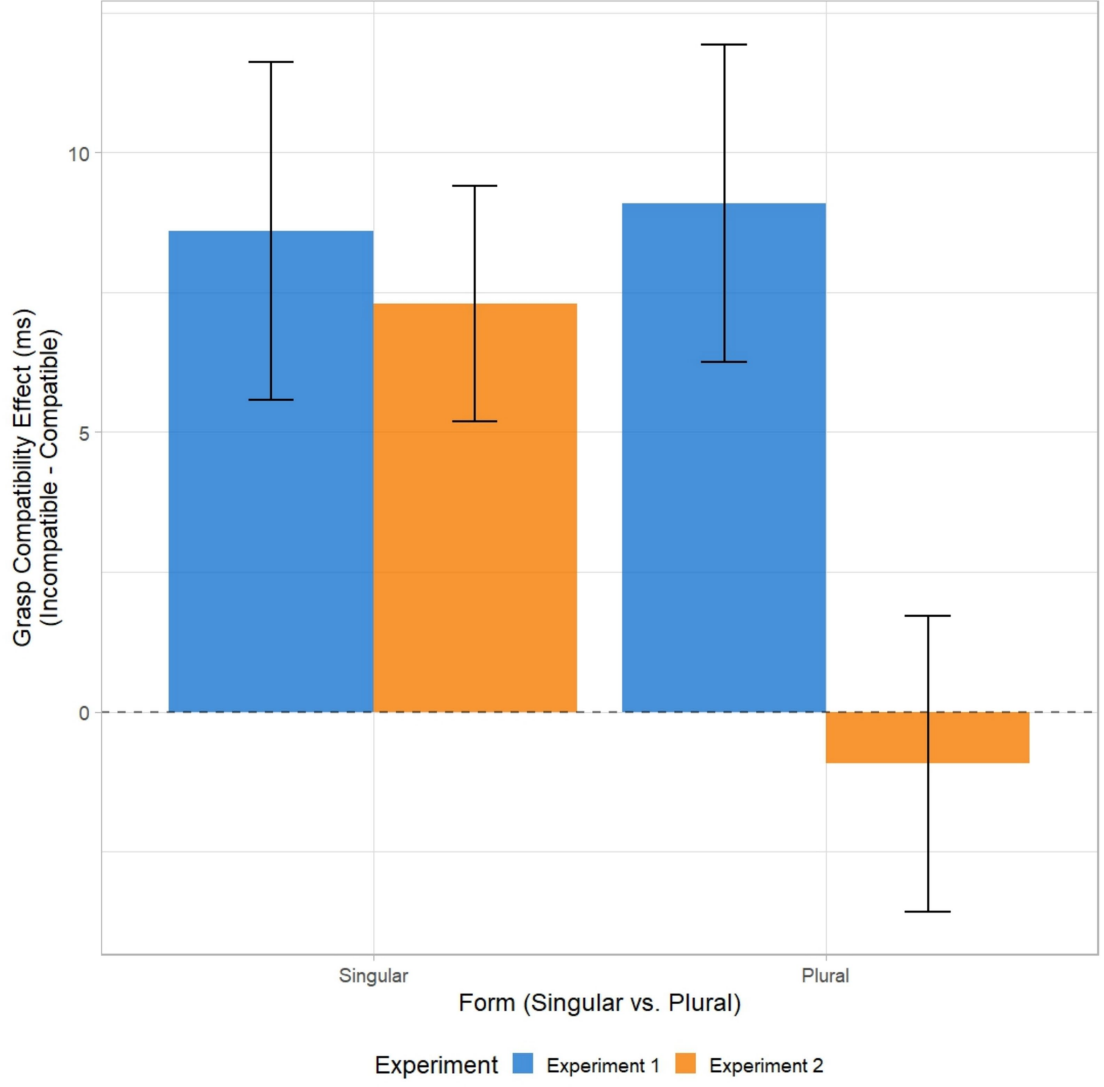
Grasp compatibility effects in Experiment 1 and 2. For each participant, GCE was calculated after transforming back mean log RTs (i.e., geometric means) and subtracting compatible RTs from incompatible ones. Error bars represent the standard error of the means (SEM).

Reaction times: Two hundred twenty-two data points were removed across all subjects from the dataset as likely anticipatory responses (0.9%). Box-Cox transformation results in a Lambda = −0.2. Accordingly, the logarithmic transformation of RTs was chosen. Outliers’ detection procedure produced a loss of 1.4% (325 datapoints) of the data. Transformed RTs were submitted to LMM. The model revealed the main effect of Grasp-Compatibility [*t*(47) = |2.819|, *p* = 0.00482; η^2^p = 0.012], with faster responses on compatible (M = 667 ms; SD = 143.76, SE = 1.40) than incompatible trials (M = 675; SD = 107.54; SEM = 23.34, see Figure 2). The main effect of Form [*t*(47) = |0.557|, *p* = 0.58] and its interaction with Grasp-Compatibility [*t*(47) = 0.086, *p* = 0.93; GCE: singular = 8 ms, plural = 10 ms] were not significant.

Movement times: Also for MTs data Box-Cox procedure showed lambda = 0. Accordingly, MTs were transformed into logarithmic. The MTs model showed a marginally reliable main effect of Grasp-Compatibility [*t*(47) = |1.810|, *p* = 0.0703] with faster response for compatible trials (M = 382; SD = 144.47; SE = 21.00) than incompatible ones (M = 387; SD = 148.32; SE = 21.64). Neither the main effect of Form nor its interaction revealed reliable effects on participants’ performance (ts < 0.9, ps > 0.35).

### Discussion

The results of Experiment 1 showed a reliable GCE, confirming that graspable objects are able to evoke motor programs consistent with how we typically interact with those objects, in line with previous results (Bub and Masson, 2012; Garofalo et al., 2021; Marino et al., 2013; Tucker and Ellis, 2004). Participants responded faster when the required grip (precision or power) matched the grip typically used to manipulate the object denoted by the noun (e.g., faster precision grip responses to “nail” than to “bottle; compatible trials), and required additional time to respond when the required grip did not match with grip evoked by the object noun (incompatible trials). This finding aligns with embodied cognition theories proposing that language comprehension involves simulation of sensorimotor experiences associated with word referents (Barsalou, 2008; Glenberg and Gallese, 2012).

However, this sensorimotor simulation was not modulated by grammatical number: similar GCEs emerged for both singular and plural forms of object nouns. A possible explanation for this result is that the sensorimotor processing of graspable object nouns primarily responds to the lexical root of the noun rather than to its full inflected form. Under this hypothesis, both “cherry” and “cherries” activate the same motor representation because they share the morphological stem {cherr-}. Accordingly, the plural morpheme {−ies} could be processed separately or even entirely ignored, failing to contribute to the sensorimotor simulation. This interpretation builds on Borghi and Riggio’s (2009, 2015) distinction between stable and variable affordances. Stable affordances derive from invariant object properties like size and shape, while variable affordances depend on context-dependent features like spatial location that require continuous updating. Borghi and Riggio (2015) proposed that language automatically encodes mainly stable affordances. From this perspective, numerosity constitutes a variable affordance: the word “cherry” activates the prototypical precision grip for a single cherry because this represents a stable affordance, while the plural morpheme does not override this motor representation since quantity is treated as variable affordance rather than as a stable one. However, Garofalo et al. (2024a) showed that the GCE evoked by object nouns was modulated by contextual features (such as agent-object distance) when made explicit through adjectives, suggesting that while nouns evoke sensorimotor simulations based on stable affordances, additional linguistic context can dynamically update these sensorimotor representations.

Hence, to further investigate whether the explicit quantification of object nouns could modulate the GCE we conducted Experiment 2. In this second study, we enhanced the sense of numerosity by pairing object nouns with explicit quantifiers, that are “uno” (one) vs. “tanti” (many). If the sensorimotor simulation evoked by the processing of graspable object nouns is sensitive to numerosity information but simply does not extract it from inflectional morphology, then explicit quantifiers should successfully modulate the GCE.

## Experiment 2

### Methods

#### Participants

Fifty-four right-handed volunteers (37 defined themselves as female, 17 as male; mean age = 20.2 ± 3.2 years) participated in the experiment at the University of Bologna. All had normal or corrected-to-normal vision, they were native Italian speakers and were naïve to the study’s purpose. The study was approved by the local ethics committee (approval number: 0026467).

#### Apparatus, stimuli and procedure

The experiment used the same setup as Experiment 1. We combined each object noun with a quantifier and specifically “uno” (one) or “tanti” (many), declined on the basis of the gender of the noun and presented before the noun according to Italian grammatical rules. Stimuli were presented as a string of words (e.g., “una oliva” one olive; “tante olive” many olives). Participants were required to categorize nouns as natural or artifact using precision or power reach-to-grasp movements. Half used power grip for natural objects and precision grip for artifacts; the other half followed the reverse mapping. Participants withheld grasp movements for pseudowords (catch trials).

#### Analyses and results

As in Experiment 1, RTs and MTs were analyzed using linear mixed models after Box-Cox transformation and outlier removal (>3 SD). Models included Grasp-Compatibility and Form (2 levels, “one” + singular vs. “many” + plural) as fixed effects, with Participants and Stimuli as random effects. Trial errors were analyzed using logistic regression.

Errors: The error rate data showed that participants made categorization errors on 2.0% (559 datapoints) of the total number of trials. Individual participants showed error rates ranging between 0 and 10.2% of all trials. The model did not show any reliable main effect or interaction.

Reaction times: Twenty hundred twenty-four data points were removed across all subjects from the dataset as likely anticipatory responses (0.8%). Box-Cox transformation results in Lambda = 0.4. Accordingly, the logarithmic transformation of RTs was chosen. Outliers’ detection procedure produced a loss of 1.1% (303 datapoints) of the data. Transformed RTs were submitted to LMM. The model revealed the main effect of Grasp-Compatibility [*t*(53) = |2.491|, *p* = 0.0128, η^2^p = 0.05], with faster responses on compatible (M = 667 ms; SD = 143.76, SE = 1.40) than incompatible trials (M = 675; SD = 107.54; SEM = 23.34). The main effect of Form did not reach the significance [*t*(53) = 0.378, *p* = 0.705]. Critically the interaction between Grasp-Compatibility and Form was reliable [*t*(53) = |2.117|, *p* = 0.034, η^2^p = 0.07]. In detail, this interaction showed that the GCE emerged with object noun paired with quantifier denoting a single unit (“one”), with faster response for compatible trials (M = 632, SD = 202.27, SE = 27.78) as compared to incompatible ones (M = 637, SD = 199.83, SE = 27.45; *z* = −3.27, *p* < 0.001). In contrast no GCE emerged for object nouns paired with “many” (Mcomp = 634, SD = 200.11 SE = 27.49; Mincomp = 633, SD = 203.42, SE = 27.94; *z* = 0.28, *p* = 0.78).

Movement times: Box-Cox procedure showed lambda = 0, hence MTs were transformed into logarithmic. The MTs model did not show any reliable main effects or interaction (ts < 1.50, ps > 0.13).

### Discussion

The second experiment investigated whether making numerosity information explicit through quantifiers (“one” vs. “many”) would successfully modulate the GCE by graspable object nouns. Results confirmed the presence of a reliable GCE, replicating the finding from Experiment 1. Critically, the predicted interaction between Grasp-Compatibility and Form emerged: the GCE was present when object nouns were paired with “one” but absent when paired with “many.” This finding is in line with previous evidence suggesting that only small quantities are compatible with grasping actions (Ranzini et al., 2011). Overall, our results show that explicit quantification can modulate the GCE during language comprehension. Accordingly, the different results between Experiments 1 and 2 can be understood through the theoretical framework distinguishing stable from variable affordances (Borghi and Riggio, 2015). The results of the present experiment show that explicit quantifiers transform numerosity from an implicit grammatical feature (e.g., the singular and plural morpheme) into a salient semantic property that becomes integrated into the sensorimotor simulation. The quantifier “one” makes explicit that a single object is present, allowing the activation of the precision or power grip appropriate for that specific object. Conversely, “many” explicitly signals multiple objects, fundamentally altering the feasibility of canonical grasping action, at least with one hand. In this respect, one may speculate that the absence of GCE with “many” could reflect a mismatch between task demands and the appropriate two-handed motor responses necessary to grasp multiple objects rather than the non-activation of the sensorimotor simulation per se.

## General discussion

The present study investigated whether object numerosity influences the sensorimotor programs automatically activated during language processing. Across two experiments using a grasp-compatibility task, participants categorized graspable object nouns as natural or artificial by performing precision or power reach-to-grasp movements. In Experiment 1, we manipulated numerosity through grammatical numbers (singular vs. plural forms). In Experiment 2, we enhanced the salience of numerosity by pairing object nouns with explicit quantifiers (“uno”; one vs. “tanti”; many).

Results revealed reliable GCEs in both experiments, replicating previous findings that processing graspable object nouns activates motor programs consistent with typical hand-object interactions (Tucker and Ellis, 2004; Marino et al., 2014). However, the critical GCE modulation by numerosity emerged only in Experiment 2. While grammatical number (singular vs. plural) failed to affect the GCE in Experiment 1, explicit quantifiers modulated the effect in Experiment 2. In fact, the GCE was present with “one” but eliminated with “many,” suggesting that specific grasping actions are only compatible with single objects (see also Ranzini et al., 2011). One might argue that the absence of modulatory effects of singular and plural forms in Experiment 1 reflects task demands, given that processing numerosity was not relevant for task. However, singular or plural form was equally task-irrelevant in Experiment 2, yet modulation was observed. This different pattern of results between experiments rules out the possibility that the lack of modulation in Experiment 1 was related to the task demands.

As a whole, the results demonstrate that the motor system is sensitive to numerosity during language comprehension, but only when this information is made sufficiently explicit through semantic quantifiers rather than grammatical morphology. Although a few previous studies have shown that grammatical numbers (singular vs. plural forms) can elicit compatibility effects similar to those observed with numerical stimuli (Roettger and Domahs, 2015; Lachmair et al., 2018), no modulatory effects of grammatical numbers were observed in our grasp-compatibility task. These findings provide novel evidence for the embodied nature of language comprehension while simultaneously revealing important constraints on sensorimotor simulation processes.

From the perspective of embodied cognition theories (Barsalou, 2008; Glenberg and Gallese, 2012), language understanding involves reactivating sensorimotor experiences associated with word referents. Our results support this view: reading object nouns automatically activates motor representations of canonical grasping actions associated with those objects. However, our data also showed that not all linguistic information equally influences these sensorimotor simulations. The contrast between experiments suggests that sensorimotor integration during language processing operates selectively, privileging explicit semantic content over implicit grammatical features. This pattern may also reflect a difference in the way singular and plural referents are conceptually represented. Singular expressions such as “one olive” evoke a concrete and individuated sensorimotor representation that directly maps onto a specific grasping action, thereby facilitating the activation of the corresponding motor program. In contrast, plural or quantified expressions like “many olives” refer to a set of objects without a clear, manipulable target, eliciting a more abstract representation that does not afford a precise motor plan. Consequently, the motor system may be engaged only when the linguistic input denotes a single, concrete entity that can be acted upon.

The theoretical framework distinguishing stable from variable affordances (Borghi and Riggio, 2009, 2015) provides a coherent account of these findings. According to this framework, stable affordances derive from relatively invariant object properties like size and shape, which are encoded in long-term semantic memory and automatically activated during object recognition. Variable affordances, in contrast, arise from context-dependent features such as spatial location, or critically for our study, quantity. Borghi and Riggio (2015) propose that language comprehension primarily recruits stable affordances while largely discounting variable ones unless they are made explicit. In Experiment 1, the plural morpheme may have been processed as a grammatical feature without sufficient semantic weight to override the prototypical motor schema stored with the object concept. Following this account, the quantifier “many” explicitly signaled a configuration (multiple objects) for which the stored motor program is inappropriate: grasping multiple apples requires qualitatively different actions that cannot be captured by the precision or power grip associated with a single object.

This interpretation aligns with recent findings by Garofalo et al. (2024a), who demonstrated that spatial adjectives (“near” vs. “far”) modulate the GCE by making spatial location explicit. Together, these studies suggest that while lexical representations of objects primarily encode stable affordances, additional linguistic elements can dynamically update sensorimotor simulations when they have sufficient semantic salience and pragmatic relevance. This view is also consistent with a wider body of work showing bidirectional connections between numerical magnitude and action. Previous research demonstrated that processing symbolic numbers automatically primes grip aperture (Moretto and di Pellegrino, 2008; Lindemann et al., 2007), and that perceiving graspable objects can interfere with numerical processing (Ranzini et al., 2011). Our findings extend this evidence by showing that during the processing of verbal stimuli, quantifiers denoting multiple objects reduce the possibility to interact with an object in a canonical way.

The grounded, embodied, and situated (GES) framework (Fischer, 2012) proposes that numerical and linguistic representations are grounded in sensorimotor experiences and situated in spatial and actional contexts. In line with this hypothesis, our results indicate that numbers, words, and actions share representational resources within a generalized magnitude system (Walsh, 2003; Bueti and Walsh, 2009). However, the present findings also suggest that the recruitment of these shared resources during language comprehension depends critically on how numerosity information is linguistically encoded. In other words, the recruitment of the sensorimotor system during language understanding appears to be flexible and context-dependent, as recently proposed (Cuccio et al., 2014; Kemmerer, 2022; Mazzuca et al., 2021; Willems and Casasanto, 2011).

In conclusion, the present findings demonstrate that object numerosity can modulate the sensorimotor simulation evoked by graspable object nouns, but only when numerosity is made explicit through semantic quantifiers rather than grammatical morphology, at least in Italian. This pattern reveals that embodied language comprehension operates under specific constraints. In the context of the grasp-compatibility task, sensorimotor simulation privileges stable affordances encoded in lexical representations, updating them only when additional linguistic elements carry sufficient information and pragmatic relevance. Future research broadening the different types of response required by the task and including measures more sensitive to implicit processing and covert motor activation may help to clarify whether grammatical number influences motor simulation under different conditions. Furthermore, it is worth noting that the present study was focused on the Italian language. However, other languages, such as Arabic for instance, are characterized by a different use of quantifiers. Therefore, another intriguing avenue for future studies concerns the cross-linguistic comparison of GCE effects and their modulations by quantifiers.

## Data Availability

The raw data supporting the conclusions of this article will be made available by the authors, without undue reservation.

## References

[ref1] Anderson M. L. (2010). Neural reuse: a fundamental organizational principle of the brain. Behav. Brain Sci. 33, 245–266. doi: 10.1017/S0140525X10000853, 20964882

[ref2] Andres M. Davare M. Pesenti M. Olivier E. Seron X. (2004). Number magnitude and grip aperture interaction. Neuroreport 15, 2773–2777, 15597052

[ref3] Badets A. Pesenti M. (2010). Creating number semantics through finger movement perception. Cognition 115, 46–53. doi: 10.1016/j.cognition.2009.11.007, 20042184

[ref4] Badets A. Pesenti M. (2011). Finger-number interaction. Exp. Psychol. 58, 287–292. doi: 10.1027/1618-3169/a000095, 21310687

[ref5] Barr D. J. Levy R. Scheepers C. Tily H. J. (2013). Random effects structure for confirmatory hypothesis testing: keep it maximal. J. Mem. Lang. 68, 255–278. doi: 10.1016/j.jml.2012.11.001, 24403724 PMC3881361

[ref6] Barsalou L. W. (2003). Situated simulation in the human conceptual system. Lang. Cogn. Process. 18, 513–562. doi: 10.1080/01690960344000026

[ref7] Barsalou L. W. (2008). Grounded cognition. Annu. Rev. Psychol. 59, 617–645. doi: 10.1146/annurev.psych.59.103006.093639, 17705682

[ref8] Bates D. Mächler M. Bolker B. Walker S. (2015). Fitting linear mixed-effects models using lme4. J. Stat. Softw. 67, 1–48. doi: 10.18637/jss.v067.i01

[ref9] Borghi A. M. Binkofski F. (2014). Words as social tools: an embodied view on abstract concepts. New York, NY: Springer.

[ref10] Borghi A. M. Riggio L. (2009). Sentence comprehension and simulation of object temporary, canonical and stable affordances. Brain Res. 1253, 117–128. doi: 10.1016/j.brainres.2008.11.064, 19073156

[ref11] Borghi A. M. Riggio L. (2015). Stable and variable affordances are both automatic and flexible. Front. Hum. Neurosci. 9:351. doi: 10.3389/fnhum.2015.00351, 26150778 PMC4473001

[ref12] Box G. E. P. Cox D. R. (1964). An analysis of transformations. J. R. Stat. Soc. Ser. B Stat Methodol. 26, 211–243. doi: 10.1111/j.2517-6161.1964.tb00553.x

[ref13] Bub D. N. Masson M. E. J. (2010). Grasping beer mugs: on the dynamics of alignment effects induced by handled objects. J. Exp. Psychol. Hum. Percept. Perform. 36, 341–358. doi: 10.1037/a0017606, 20364923

[ref14] Bub D. N. Masson M. E. J. (2012). On the dynamics of action representations evoked by names of manipulable objects. J. Exp. Psychol. Gen. 141, 502–517. doi: 10.1037/a0026748, 22201414

[ref15] Bueti D. Walsh V. (2009). The parietal cortex and the representation of time, space, number and other magnitudes. Philos. Trans. R. Soc. Lond. B. Biol. Sci. 364, 1831–1840. doi: 10.1098/rstb.2009.0028, 19487186 PMC2685826

[ref16] Castiello U. (1999). Mechanisms of selection for the control of hand action. Trends Cogn. Sci. 3, 264–271. doi: 10.1016/S1364-6613(99)01346-7, 10377541

[ref17] Castiello U. (2005). The neuroscience of grasping. Nat. Rev. Neurosci. 6, 726–736. doi: 10.1038/nrn1744, 16100518

[ref18] Cuccio V. Ambrosecchia M. Ferri F. Carapezza M. Lo Piparo F. Fogassi L. . (2014). How the context matters. Literal and figurative meaning in the embodied language paradigm. PLoS One 9:e115381. doi: 10.1371/journal.pone.0115381, 25531530 PMC4274021

[ref19] Ellis R. Tucker M. (2000). Micro-affordance: the potentiation of components of action by seen objects. Br. J. Psychol. 91, 451–471. doi: 10.1348/000712600161934, 11104173

[ref20] Ellis R. Tucker M. Symes E. Vainio L. (2007). Does selecting one visual object from several require inhibition of the actions associated with nonselected objects? J. Exp. Psychol. Hum. Percept. Perform. 33, 670–691. doi: 10.1037/0096-1523.33.3.670, 17563229

[ref21] Fischer M. H. (2012). A hierarchical view of grounded, embodied, and situated numerical cognition. Cogn. Process. 13, S161–S164. doi: 10.1007/s10339-012-0477-5, 22802036

[ref22] Fodor J. A. (1975). The language of thought, vol. 5. Cambridge, MA: Harvard University Press.

[ref23] Gallese V. (2003). A neuroscientific grasp of concepts: from control to representation. Philos Trans R Soc Lond B Biol Sci 358, 1231–1240. doi: 10.1098/rstb.2003.1315, 12880530 PMC1693221

[ref24] Gallese V. (2008). Mirror neurons and the social nature of language: the neural exploitation hypothesis. Soc. Neurosci. 3, 317–333. doi: 10.1080/17470910701563608, 18979384

[ref25] Gallese V. Cuccio V. (2018). The neural exploitation hypothesis and its implications for an embodied approach to language and cognition: insights from the study of action verbs processing and motor disorders in Parkinson's disease. Cortex 100, 215–225. doi: 10.1016/j.cortex.2018.01.010, 29455947

[ref26] Gallese V. Lakoff G. (2005). The brain's concepts: the role of the sensory-motor system in conceptual knowledge. Cogn. Neuropsychol. 22, 455–479. doi: 10.1080/02643290442000310, 21038261

[ref27] Garofalo G. Gherri E. Riggio L. (2024b). Syntax matters in shaping sensorimotor activation driven by nouns. Mem. Cogn. 52, 285–301. doi: 10.3758/s13421-023-01460-0, 37672153

[ref28] Garofalo G. Marino B. F. M. Bellelli S. Riggio L. (2021). Adjectives modulate sensorimotor activation driven by nouns. Cogn. Sci. 45, e12953–e12936. doi: 10.1111/cogs.12953, 33755244

[ref29] Garofalo G. Riggio L. Bianchini F. Gherri E. 2024a The space of words: semantic integration of space-related words in noun sensorimotor processing Manuscript submitted for publication 10.1007/s00426-026-02326-1 PMC13452750 42570113

[ref30] Gibson J. J. (1979). The ecological approach to visual perception. Boston, MA: Houghton Mifflin.

[ref31] Glenberg A. M. Gallese V. (2012). Action-based language: a theory of language acquisition, comprehension, and production. Cortex 48, 905–922. doi: 10.1016/j.cortex.2011.04.010, 21601842

[ref32] Glover S. (2004). Separate visual representations in the planning and control of action. Behav. Brain Sci. 27, 3–24. doi: 10.1017/S0140525X04000020, 15481943

[ref33] Jeannerod M. (1984). The timing of natural prehension movements. J. Mot. Behav. 16, 235–254. doi: 10.1080/00222895.1984.10735319, 15151851

[ref34] Kemmerer D. (2022). Grounded cognition entails linguistic relativity: a neglected implication of a major semantic theory. Top. Cogn. Sci. 15, 615–647. doi: 10.1111/tops.12628, 36228603

[ref35] Klein Entink R. H. Fox J.-P. van der Linden W. J. (2009). A multivariate multilevel approach to the modeling of accuracy and speed of test takers. Psychometrika 74, 21–48. doi: 10.1007/s11336-008-9075-y, 20037635 PMC2792348

[ref36] Lachmair M. Dudschig C. de la Vega I. Kaup B. (2014). Relating numeric cognition and language processing: do numbers and words share a common representational platform? Acta Psychol. 148, 107–114. doi: 10.1016/j.actpsy.2013.12.004, 24509403

[ref37] Lachmair M. Ruiz Fernandez S. Gerjets P. (2018). Does grammatical number influence the semantic priming between number cues and words related to vertical space? An investigation using virtual reality. Front. Psychol. 9:573. doi: 10.3389/fpsyg.2018.00573, 29731734 PMC5921996

[ref38] Lindemann O. Abolafia J. A. Girardi G. Bekkering H. (2007). Getting a grip on numbers: numerical magnitude priming in object grasping. J. Exp. Psychol. Hum. Percept. Perform. 33, 1400–1409. doi: 10.1037/0096-1523.33.6.1400, 18085952

[ref39] Makris S. Hadar A. A. Yarrow K. (2011). Viewing objects and planning actions: on the potentiation of grasping behaviours by visual objects. Brain Cogn. 77, 257–264. doi: 10.1016/j.bandc.2011.08.002, 21903319

[ref40] Marino B. F. M. Gough P. M. Gallese V. Riggio L. Buccino G. (2013). How the motor system handles nouns: a behavioral study. Psychol. Res. 77, 64–73. doi: 10.1007/s00426-011-0371-2, 21879354

[ref41] Marino B. F. M. Sirianni M. Volta R. D. Magliocco F. Silipo F. Quattrone A. . (2014). Viewing photos and reading nouns of natural graspable objects similarly modulate motor responses. Front. Hum. Neurosci. 8:968. doi: 10.3389/fnhum.2014.00968, 25538596 PMC4255516

[ref42] Mazzuca C. Fini C. Michalland A. H. Falcinelli I. Da Rold F. Tummolini L. . (2021). From affordances to abstract words: the flexibility of sensorimotor grounding. Brain Sci. 11:1304. doi: 10.3390/brainsci11101304, 34679369 PMC8534254

[ref43] Moretto G. di Pellegrino G. (2008). Grasping numbers. Exp. Brain Res. 188, 505–515. doi: 10.1007/s00221-008-1386-9, 18427791

[ref44] Namdar G. Tzelgov J. Algom D. Ganel T. (2014). Grasping numbers: evidence for automatic influence of numerical magnitude on grip aperture. Psychon. Bull. Rev. 21, 830–835. doi: 10.3758/s13423-013-0550-9, 24222367

[ref45] Oldfield R. C. (1971). The assessment and analysis of handedness: the Edinburgh inventory. Neuropsychologia 9, 97–113. doi: 10.1016/0028-3932(71)90067-4, 5146491

[ref46] Pavese A. Buxbaum L. J. (2002). Action matters: the role of action plans and object affordances in selection for action. Vis. Cogn. 9, 559–590. doi: 10.1080/13506280143000584

[ref47] Pulvermüller F. (2002). A brain perspective on language mechanisms: from discrete neuronal ensembles to serial order. Prog. Neurobiol. 67, 85–111. doi: 10.1016/S0301-0082(02)00014-X, 12126657

[ref48] Pulvermüller F. (2018). Neurobiological mechanisms for semantic feature extraction and conceptual flexibility. Top. Cogn. Sci. 10, 590–620. doi: 10.1111/tops.12367, 30129710

[ref49] Pylyshyn Z. W. (1984). Computation and cognition. Cambridge, MA: MIT press.

[ref50] Pylyshyn Z. W. (2003). Return of the mental image: are there really pictures in the brain? Trends Cogn. Sci. 7, 113–118. doi: 10.1016/S1364-6613(03)00003-2, 12639692

[ref51] R Core Team (2022). R: A language and environment for statistical computing. Vienna, Austria: R foundation for statistical computing.

[ref52] Ranzini M. Lugli L. Anelli F. Carbone R. Nicoletti R. Borghi A. M. (2011). Graspable objects shape number processing. Front. Hum. Neurosci. 5:147. doi: 10.3389/fnhum.2011.00147, 22164141 PMC3230823

[ref53] Ranzini M. Semenza C. Zorzi M. Cutini S. (2022). Influences of hand action on the processing of symbolic numbers: a special role of pointing? PLoS One 17:e0269557. doi: 10.1371/journal.pone.0269557, 35687556 PMC9187111

[ref54] Restle F. (1970). Speed of adding and comparing numbers. J. Exp. Psychol. 83:274. doi: 10.1037/h0028573

[ref55] Roettger T. B. Domahs F. (2015). Grammatical number elicits SNARC and MARC effects as a function of task demands. Q. J. Exp. Psychol. 68, 1231–1248. doi: 10.1080/17470218.2014.979843, 25384199

[ref56] Santana E. J. De Vega M. (2013). An ERP study of motor compatibility effects in action language. Brain Res. 1526, 71–83. doi: 10.1016/j.brainres.2013.06.020, 23796780

[ref57] Symes E. Tucker M. Ellis R. Vainio L. Ottoboni G. (2008). Grasp preparation improves change detection for congruent objects. J. Exp. Psychol. Hum. Percept. Perform. 34, 854–871. doi: 10.1037/0096-1523.34.4.854, 18665731

[ref58] Taylor L. J. Zwaan R. A. (2009). Action in cognition: the case of language. Lang. Cogn. 1, 45–58. doi: 10.1515/langcog.2009.003

[ref59] Tucker M. Ellis R. (2004). Action priming by briefly presented objects. Acta Psychol. 116, 185–203. doi: 10.1016/j.actpsy.2004.01.004, 15158182

[ref60] Van Dam W. O. van Dijk M. Bekkering H. Rueschemeyer S. A. (2012). Flexibility in embodied lexical-semantic representations. Hum. Brain Mapp. 33, 2322–2333. doi: 10.1002/hbm.21365, 21976384 PMC6869997

[ref61] Varma S. Sanford E. M. Marupudi V. Shaffer O. Lea R. B. (2024). Recruitment of magnitude representations to understand graded words. Cogn. Psychol. 153:101673. doi: 10.1016/j.cogpsych.2024.101673, 39094253

[ref62] Visani E. Garofalo G. Sebastiano D. R. Duran D. Craighero L. (2022). Grasping the semantic of actions: a combined behavioral and MEG study. Front. Hum. Neurosci. 16:1008995. doi: 10.3389/fnhum.2022.1008995, 36583012 PMC9792482

[ref63] Walsh V. (2003). A theory of magnitude: common cortical metrics of time, space and quantity. Trends Cogn. Sci. 7, 483–488. doi: 10.1016/j.tics.2003.09.002, 14585444

[ref64] Willems R. M. Casasanto D. (2011). Flexibility in embodied language understanding. Front. Psychol. 2, 1–11. doi: 10.3389/fpsyg.2011.00116, 21779264 PMC3132681

